# Adaptation modulates effective connectivity and network stability

**DOI:** 10.3389/fncom.2026.1761735

**Published:** 2026-04-10

**Authors:** Thomas J. Richner, Martynas Dervinis, Brian Nils Lundstrom

**Affiliations:** Department of Neurology, Mayo Clinic, Rochester, MN, United States

**Keywords:** adaptation, edge of chaos, recurrent neural networks, spike frequency adaptation, short-term synaptic depression, effective connectivity, random matrix theory, excitation-inhibition balance

## Abstract

The brain is a highly recurrent, nonlinear network hypothesized to remain near the edge of chaos for optimal performance. Excitation and inhibition must be balanced precisely within every neuron to ensure a consistent level of dynamical stability and rich dynamics during transition to chaos. However, analysis of biologically realistic synaptic weight matrices suggests that sparsity and low-dimensional structure interact such that there is no known synaptic balancing rule that constrains the stability (i.e., eigenvalues) of the network while also preserving computationally useful, low-dimensional structure. Further, even if a network were well-balanced, external stimuli interact with the nonlinear activation functions to unbalance the network in real time. Therefore, the brain must utilize dynamic, rather than static, mechanisms to actively regulate its level of stability. We propose that two specific adaptation mechanisms, spike frequency adaptation (SFA) and short-term synaptic depression (STD), continuously modulate the effective connectivity, keeping the brain near the edge of chaos and reducing dynamical fluctuations caused by stimuli. This theoretical framework links intrinsic and synaptic negative feedback mechanisms to network-level dynamics. This offers an explanation of why data-driven modeling of human brain signals, an exciting and useful method in epilepsy and anesthesiology research, seems to require linear time-varying (LTV) models which are refit every half second: difficult to observe adaptation processes interact with nonlinearities to make connectivity effectively dynamic at the macroelectrode scale. We suggest that compromised adaptation may underlie neurological conditions characterized by altered excitability, and that targeted brain stimulation could be used to probe the regulatory action of adaptation.

## 1 Introduction

The brain is a highly recurrent, sparse, non-linear network of neurons. The dynamics of the brain, the extent to which signals are amplified or suppressed, must be closely regulated. However, the cortex has about 5-fold more excitatory-excitatory (E-E) reciprocal connections than would be expected by chance (Campagnola et al., 2022). These reciprocal E-E connections create positive feedback and, combined with nonlinearity, can lead to chaos (Lorenz, 1963; May, 1976). Too much chaos, or too much stability, combined with stochastic noise can degrade the decodability of information (James et al., 2014). Therefore, it is hypothesized that the brain operates near the edge of chaos for optimal information storage and processing capacity (Bertschinger and Natschläger, 2004; Boedecker et al., 2012; Ganguli et al., 2008; Langton, 1990; Legenstein and Maass, 2007).

Data-driven dynamical models have the potential to quantify the level of stability or chaos in the brain and how it fluctuates. Several groups using vector autoregression (VAR) or linear time-varying (LTV) models have found that during wakefulness, eigenvalues of the effective connectivity cluster near the stability boundary and that anesthesia reduces the number of eigenvalues near the boundary (Alonso et al., 2014, 2014; Dávila et al., 2025; Solovey et al., 2015), supporting the hypothesis that the brain operates near the edge of chaos, and that perturbations that alter its dynamical properties lead to loss of function.

A range of physiologic mechanisms regulate neuron activity and synaptic distributions over extended timescales (Hennequin et al., 2017; Marder and Goaillard, 2006; Turrigiano, 2012; Wen and Turrigiano, 2024). However, even if the structural (i.e., synaptic) connectivity is well-regulated, the effective connectivity is potentially susceptible to rapid fluctuations. For this paper, we define effective connectivity as the first-order, time-varying, network of causal neural interactions. Due to each neuron’s rectifying frequency-current (F-I) response function (i.e., activation function), and the fact that most neurons have a low basal firing rate near the nonlinear transition, the subnetwork of active neurons is ever changing due to internal and external stimuli. There is no guarantee that different activated subnetworks have similar stability. Thus, structural constraints alone may be insufficient to maintain the network near the edge of chaos.

The excitability of neurons and synapses is dynamic (Abbott et al., 1997; Adrian and Zotterman, 1926; Connors and Gutnick, 1990; Dong and Hopfield, 1992; Tsodyks and Markram, 1997). Each neuron and each synapse has an internal state that depends on its recent history of input and output. For instance, spike frequency adaptation (SFA) is mediated by several mechanisms including voltage-gated potassium channels, calcium-activated potassium channels, and inactivation of sodium channels (Benda and Herz, 2003; Marom and Marder, 2023), so the cytosolic calcium state determines, in part, how fast a neuron fires in response to a sustained stimulus. At the synapse level, synapses are dynamic due to short-term synaptic plasticity, such as depression. Short-term synaptic depression (STD) due to depletion depends on the state of vesicles ready to be released (Abbott et al., 1997; Tsodyks and Markram, 1997). These internal states are difficult to observe (i.e., latent) during extracellular recordings.

At the macroscopic scale of local field potentials (LFPs) or stereo-electroencephalograph (SEEG), neuronal excitability, synaptic connectivity, and synaptic dynamics are mixed non-linearly, yielding effective connectivity. While homeostatic plasticity mechanisms adjust slowly, adaptation operates quickly, over timescales similar to that of sensory stimuli (from tens of milliseconds to tens of seconds). Therefore, we propose that adaptation compensates for the stability fluctuations caused by stimuli, dynamically modifying effective connectivity to preserve a consistent dynamical regime.

We define adaptation broadly to include synaptic, neuronal, and microcircuit mechanisms that cause neurons to reduce their output during sustained input. Here, we focus on STD and SFA. We suggest that adaptation continuously modifies effective connectivity in the brain toward a homeostatic setpoint near the edge of chaos. Here, we (1) review the excitation-inhibition balance theoretically in the absence of adaptation, and (2) propose how adaptation can be modeled and incorporated into effective connectivity. In the discussion, we relate this framework to current data-driven modeling approaches.

## 2 Stability of networks without adaptation

To understand the necessity of adaptation for maintaining the brain near the edge of chaos, we first examine the stability of networks with static connections. We analyze local stability using a linear time-invariant (LTI) approximation of a recurrent network, $\frac{d\,x}{d\,t}=W\,x$. The stability of this system is governed by the eigenvalues λ of the connectivity matrix *W* (Lyapunov, 1892). Each complex eigenvalue describes an oscillatory mode of the network, where the real component, *Re*(λ), determines the mode’s exponential growth rate. Stability is ensured only if all modes decay (i.e., *Re*(λ) < 0 for all λ). While this linear framework defines the threshold for instability, the crossing of an eigenvalue into the right half plane, nonlinearities are required to bound this growth and support the complex attractors characteristic of neural computation.

Neurons exhibit a non-linear, typically sigmoidal, response to input current, a property captured by the activation function ϕ in Hopfield networks (Hopfield, 1982, 1984):


$${\dot{x}}_{i}\,\left(t\right)=-x_{i}\,\left(t\right)+\underset{j}{\sum }W_{i\,j}\,\phi \,\left(x_{j}\,\left(t\right)\right)$$ (1)

With symmetric connectivity (*W*_*ij*_ = *W*_*ji*_), these systems relax to stable attractors, but when connectivity is asymmetric, these networks can exhibit deterministic chaos (Sompolinsky et al., 1988). Like eigenvalues for LTI systems, Lyapunov exponents quantify the exponential rate of expansion or contraction in nonlinear systems. The evolution of a small perturbation δ*x* is $\dot{\delta \,x}=J\,\left(x\,\left(t\right)\right)\,\delta \,x$ where *J* is the Jacobian. The expansion and rotation of the system are captured by the fundamental matrix Φ(*t*), the time-varying state-transition matrix of the linearized perturbation dynamics, which evolves as


$$\dot{\Phi }\,\left(t\right)=J\,\left(x\,\left(t\right)\right)\,\Phi \,\left(t\right)\;\mathrm{with}\;\Phi \,\left(0\right)=I$$

The Lyapunov exponents *λ*^*Lyp*^ are determined by the singular values σ_*i*_(*t*) of Φ(*t*), defined as $\lambda_{i}^{L\,y\,p}=\lim_{t\to \infty }\frac{1}{t}\,\ln\sigma_{i}\,\left(t\right)$. While a positive largest Lyapunov exponent $\lambda_{1}^{L\,y\,p}$ indicates chaos, if the network is resting at a fixed point, then λiL⁢y⁢p=Re⁢(λie⁢i⁢g). Thus, near a fixed point, we can simply analyze the eigenvalues $\lambda_{i}^{e\,i\,g}$ of the linearized Jacobian. Further, if we assume *ϕ*′ ≈ 1, then we can utilize random matrix theory (which requires matrix elements to be independent and identically distributed) to understand the transition to chaos of large randomly connected networks via eigen analysis.

The theoretical understanding of stability in large, complex systems has evolved significantly since early investigations into ecological and neural networks. Countering the intuition that larger complex systems are more stable (MacArthur, 1955), early simulations showed that large systems become unstable beyond a critical connection density (Gardner and Ashby, 1970). May drew on random matrix theory (Ginibre, 1965), to show that larger systems must have weaker connections, or increased self-negative feedback, to avoid instability (May, 1972). For the network defined in Equation 1, if ϕ (*x*) = *x*, the Jacobian is


J=W-I

**FIGURE 1:**
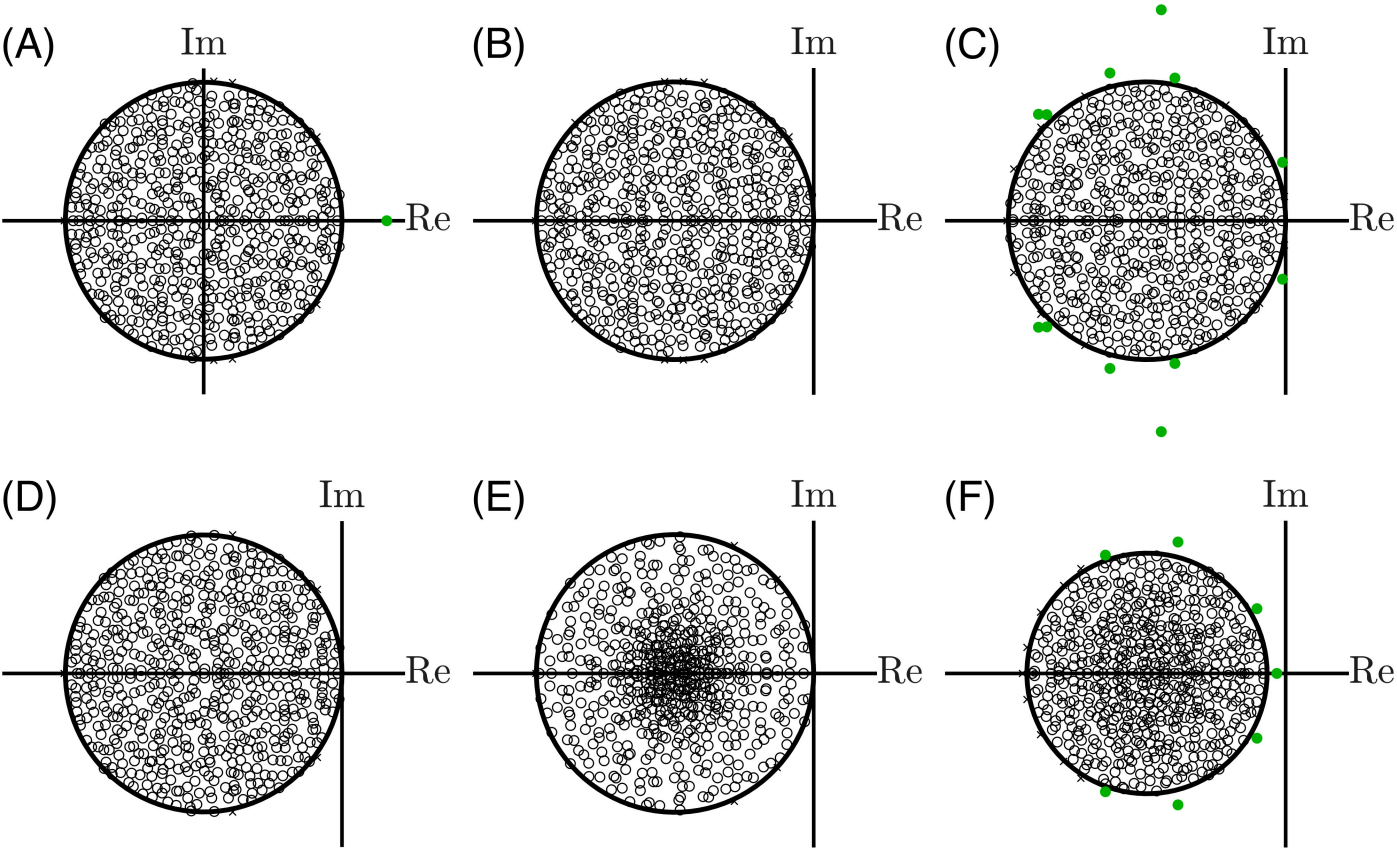
Random matrix theory can be used to understand the necessary conditions for stability in LTI networks. **(A)** If the elements of *W* are independent and identically distributed, the bulk of eigenvalues will reside within a disk on the complex plane, the circular law. However, in this example, the mean is slightly positive, creating an outlier eigenvalue in green. **(B)** Negative feedback along the main diagonal shifts all eigenvalues left such that this network is on the edge of stability. Note that the expected value is zero for B-F **(C)** If Dale’s law is implemented such that excitatory and inhibitory neurons have different mean synaptic weights, outlier eigenvalues will arise (green), even though the synaptic weights average to zero across the network. These outliers can destabilize the system as they cross the imaginary axis. **(D)** These outliers can be reined in by ensuring each neuron receives precisely balanced excitatory and inhibitory synaptic weights. **(E)** If the excitatory and inhibitory synaptic weight distributions have different standard deviations, the eigenvalues will be concentrated near the center. **(F)** Most importantly, if the connectivity matrix is sparse (50% in this example), balancing each neuron’s input weights no longer constrains the outlier eigenvalues (green) without also abolishing the low-rank structure of the matrix (Harris et al., 2023), motivating the need for adaptation.

Consequently, if the eigenvalues of *W* are λ, the eigenvalues of *J* are λ − 1. Stability therefore requires all eigenvalues of *J* to have real part less than zero and all eigenvalues of *W* to have a real part less than one. This implies that the entire eigenspectrum of *J* can be shifted by manipulating the main diagonal (Figure 1B). Importantly, the eigenvalues of an *N* × *N* random matrix with zero mean μ and standard deviation σ are uniformly distributed within a disk of radius $R\approx \sigma \,\sqrt{N}$, the circular law (Girko, 1985; Figure 1A), which was mathematically proven for a wide range of distributions (Tao and Vu, 2008). However, a non-zero mean μ creates a real outlier eigenvalue at approximately μ*N* (Figure 1A). As long as μ is negative, inhibitory dominance, stability will be governed by the bulk of eigenvalues within the disk (Ipsen and Peterson, 2020).

Biological neural networks violate the independent and identically distributed assumption of standard random matrix theory due to Dale’s law, where neurons have either excitatory or inhibitory outputs (Dale, 1935). When Dale’s law is applied, the bulk spectrum adheres to the circular law, but the dissimilar mean connection weights of excitatory and inhibitory populations create outlier eigenvalues scattered outside the disk (Rajan and Abbott, 2006; Figure 1C). This structure can be modeled as a random matrix *A* perturbed by a low-rank term *u*^*vT*^ that sets the mean synaptic strength of each neuron population. More generally, low-rank synaptic structures allow the embedding of computations and stable trajectories within a chaotic background (Mastrogiuseppe and Ostojic, 2018). However, if not constrained, the outliers will lead the transition to chaos (ahead of the bulk spectrum), dramatically reducing the number of unstable equilibria and the complexity of dynamics (Ipsen and Peterson, 2020). To suppress these outliers, the rows of the connectivity matrix must sum to zero (Rajan and Abbott, 2006; Figure 1D). This means excitatory and inhibitory weights must be balanced within the dendrite of every neuron. Further, if the neuron populations have different weight standard deviations, the eigenvalue distribution concentrates near the center, away from the edge of stability (Aljadeff et al., 2015a, b; Rajan and Abbott, 2006; Figure 1E), decreasing the dynamical complexity during the transition to chaos by reducing the number of unstable modes (Ipsen and Peterson, 2020) that are essential for a rich information storage capacity and computation (Bertschinger and Natschläger, 2004; Boedecker et al., 2012; Ganguli et al., 2008; Langton, 1990; Legenstein and Maass, 2007).

The circular law extends to sparse random networks (Tao and Vu, 2008) even with vanishing low connection density (Götze and Tikhomirov, 2010). For a balanced network with *N* neurons, connection density *d*, and synaptic weights with standard deviation σ, the spectral radius is $R\approx \sigma \,\sqrt{N\,d}$. An algebraic consequence is that if the expected indegree *K* = *Nd* is held constant as network size *N* increases, the spectral radius remains invariant to network size. This offers a theoretical explanation for why mouse and human cortical neurons have a similar number of synapses per neuron even though human neurons have much bigger dendritic trees (Loomba et al., 2022).

However, a fundamental tension exists between the sparsity of biological neural networks and the structural differences between excitatory and inhibitory populations (Harris et al., 2023; Figure 1F). To prevent unstable eigenvalue outliers, the input synaptic weights to each neuron must be precisely balanced. But this balancing act becomes impossible when networks are both sparse and have distinct excitatory/inhibitory populations. Attempting to balance inputs globally erases meaningful distinctions between these populations. Conversely, attempting to balance only the random connectivity fails because sparsity renders the population structure a new source of randomness that escapes control. Harris et al. (2023) conclude that finding “an appropriate condition to constrain these eigenvalues and preserve inhibitory dominance (imbalance) remains an open problem.”

We propose that the condition that constrains outlier eigenvalues might not be structural but dynamic: physiological adaptation mechanisms, operating on faster timescales than structural plasticity, may actively rein in these outliers to ensure a consistent level of stability or chaos. We further point out that even if a network is well-balanced structurally, external input can interact with the nonlinear activation function to create further imbalance. The solution may involve effective connectivity that is not fixed in time but rather evolves dynamically due to interactions between time-varying external inputs, nonlinear activation functions, and adaptation.

## 3 Adaptation modulates effective connectivity

Physiological adaptation mechanisms operate across a range of timescales, dynamically controlling the input-output relationship of neural circuits at both the somatic and synaptic levels (Gjorgjieva et al., 2016). These mechanisms continuously adjust excitability based on their history of activity. We focus on two primary forms of adaptation: spike frequency adaptation (SFA) and short-term synaptic depression (STD), although other mechanisms or motifs are similar (Tripp and Eliasmith, 2010). By modeling these processes alongside structural connectivity, we can derive a time-varying effective connectivity that describes the instantaneous stability of the network.

Spike frequency adaptation is a prevalent property of neurons characterized by a reduction in firing rate during sustained stimulation, a phenomenon described a century ago (Adrian and Zotterman, 1926). Over half of human cortical neurons and nearly a third of mouse cortical neurons exhibit SFA (Gouwens et al., 2019; Salaj et al., 2021). SFA is essential for efficient coding, allowing neural circuits to resolve ambiguities in sensory input and optimize information transmission (Fairhall et al., 2001; Gutierrez and Denève, 2019; Salaj et al., 2021), and can be modeled with additional first-order differential equations (Benda and Herz, 2003; Lundstrom, 2015). And while often modeled with a single timescale, SFA in neocortical pyramidal neurons exhibits multiple timescales of adaptation, a property that approximates fractional-order differentiation of the input (Lundstrom et al., 2008, 2010). Each timescale *k* can be modeled using a first-order differential equation, where the adaptation state variable *a*_*ik*_ for neuron *i* acts as a negative feedback current that grows with neuronal activity *r_i_*:


$${\dot{a}}_{i\,k}=\frac{-a_{i\,k}+r_{i}}{\tau_{k}}$$

These adaptation variables combine to alter the firing rate, effectively shifting the neuron’s operating point on its activation function (Lundstrom, 2015; Lundstrom et al., 2008; Lundstrom and Richner, 2023; Tripp and Eliasmith, 2010). In the context of network dynamics, SFA functions as an adaptive bias, acting as a time-dependent negative self-connection that tends to stabilize the system.

At the synaptic level, short-term plasticity dynamically regulates the strength of connections based on presynaptic activity. STD serves as a gain control mechanism (Abbott et al., 1997; Tsodyks and Markram, 1997). STD is often described using a resource depletion model, where the available pool of neurotransmitter (represented by the fraction *b_i_*) decreases with each release event (dependent on firing rate *r_i_*) and recovers with a characteristic time constant:


$${\dot{b}}_{i}=\frac{1-b_{i}}{\tau_{r\,e\,c}}-\frac{b_{i}\,r_{i}}{\tau_{r\,e\,l}}$$

Here, τ_*rel*_ and τ_*rec*_ represent the release and recovery time constants, respectively (Abbott et al., 1997; Tsodyks and Markram, 1997). This phenomenology has led to the concept of the “dynamic synapse,” which fundamentally expands the computational power of neural connections (Choquet and Triller, 2013; Liaw and Berger, 1996). In recurrent networks, dynamic synapses induce rich behaviors, including the emergence of chaos (Dror and Tsodyks, 2000). STD introduces a multiplicative nonlinearity into the network equations, acting as an adaptive synaptic weight (scaling the columns of *W* by *b_j_*). While SFA alone is insufficient to stabilize networks with supralinear neuronal activation functions, STD effectively quenches runaway dynamics by rapidly reducing gain in response to transient bursts (Wu and Zenke, 2021). Because the multiplicative nonlinearity of STD can drive synaptic weights toward zero during high activity, it provides a qualitatively stronger stabilizing mechanism than the subtractive feedback of SFA. SFA and STD can interact in interesting nonlinear ways and may underlie findings related to changes of interictal low-frequency EEG activity that may localize seizure onset (Lundstrom et al., 2021; Lundstrom and Richner, 2023). We hypothesize that multiple timescale SFA helps suppress peaks in the transfer function *H*_∞_ that would otherwise be present with single timescale SFA or STD, thereby helping to position a diverse range of eigenvalues near the edge of chaos, improving complexity.

To illustrate the interaction of multiple timescale adaptation, nonlinear activation functions, and external input, consider a recurrent neural network with synaptic connectivity *W* incorporating short-term synaptic depression and multiple timescale SFA. *x_i_* is analogous to the dendritic potential of the neuron *i*, *u_i_* is the external stimulus, ϕ is the neuron’s sigmoidal activation function (e.g., logistic function), *r_i_* is the firing rate, and *b_i_* is the fraction of available synaptic vesicles due to STD with release and recovery time constants τ_*rel*_ and τ_*rec*_. The neuron has *K* SFA timescales with states *a*_*ik*_, each evolving with a time constant τ_*k*_, and *a*_0_*i*__ represents a constant shift within the activation function to set the baseline firing rate near the lower inflection point of the sigmoid. τ_*d*_ is the dendritic time constant. The system is governed by:


$$\begin{array}{l} \begin{array}{c} \;{\dot{x}}_{i}=\frac{-x_{i}+u_{i}+\sum_{j=1}^{J}w_{ij}b_{j}r_{j}}{\tau_{d}} \end{array}\\ \;r_{i}=\phi \left(x_{i}-a_{0_{i}}-c\sum_{k=1}^{K}a_{ik}\right)\\ \;{\dot{a}}_{ik}=\frac{-a_{ik}+r_{i}}{\tau_{k}}\\ \begin{array}{c} \begin{array}{c} \;{\dot{b}}_{i}=\frac{1-b_{i}}{\tau_{rec}}-\frac{b_{i}r_{i}}{\tau_{rel}} \end{array} \end{array} \end{array}$$ (2)

In this framework, the effective connectivity *J*_*eff*_ of the network is not static but evolves as a function of the state variables *x*, *a*, and *b*. By linearizing the system around the current state, we derive *J*_*eff*_ as the Jacobian of the dynamics with respect to *x*. Using matrix notation where *B* = *diag*(*b*), Φ′(*h*) = *diag*(ϕ′(*h*)), and *h* = *x*_*i*_−*a*_0_*i*__$-c\,\sum_{k=1}^{K}a_{i\,k}$, we define the effective connectivity for our model (Equation 2) as:


$$\,J_{\mathrm{eff}}\,\left(x,a,b\right)=\frac{1}{\tau_{d}}\,\left(-I+W\,B\,\Phi^{{}^{\prime }}\,\left(h\right)\right)$$ (3)

**FIGURE 2:**
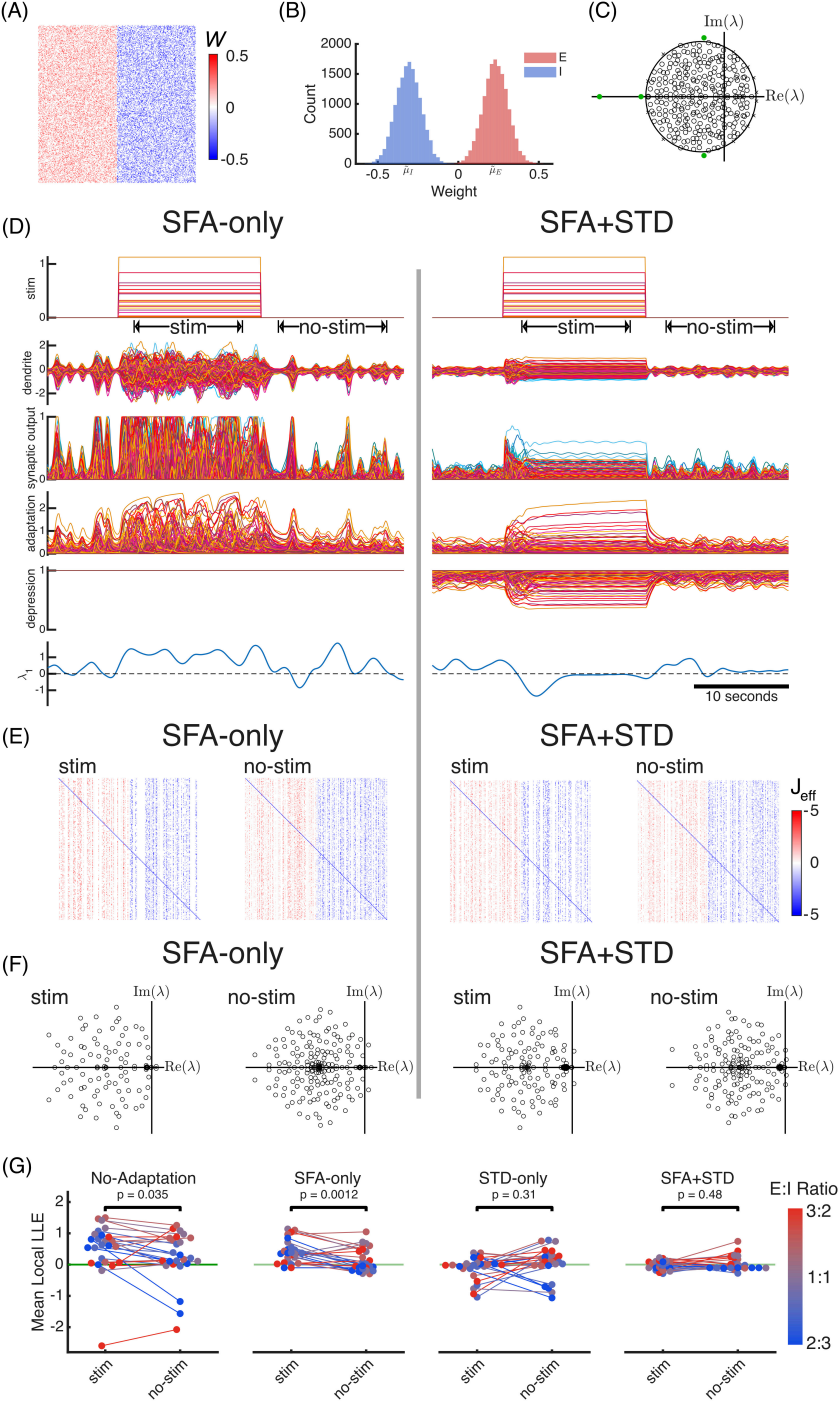
Networks with both spike frequency adaptation (SFA) and short-term synaptic depression (STD) stay near the edge of chaos, even as the stimulus changes. We simulated a network (Equation 2) with 300 neurons using a random, sparse (*d* = 1/3) connection matrix *W* **(A)** with Gaussian distributed excitatory and inhibitory weights **(B)** and with modest inhibitory dominance (${\tilde{\mu }}_{I}=\frac{4}{3}\,{\tilde{\mu }}_{E}$). The eigenspectrum **(C)** of the linearized Jacobian $J=\frac{1}{\tau_{d}}\,\left(W-I\right)$ has many unstable values right of the imaginary axis, necessitating stability promoting mechanisms. We used a hard sigmoid nonlinearity ϕ with rounded corners and range of [0, 1] to further enforce Dale’s law on synaptic output, unlike the typical *tanh*(*x*) . **(D)** Excitatory (but not inhibitory) neurons had SFA-only (left) or SFA+STD (right). A random amplitude step stimulus was applied. The SFA-only network has a higher basal local largest Lyapunov exponent $\lambda_{1}^{l\,o\,c\,a\,l}>0$, indicating chaos that fluctuates and increases during the stim period. The same network with both SFA + STD has a more consistent local largest Lyapunov exponent that reduces fluctuation amplitude while not significantly changing mean value during the stim period after the stimulus onset initial transient. We computed the finite time largest Lyapunov exponent and local largest Lyapunov exponent using Benettin’s rescaling shadow trace method (Benettin et al., 1976). **(E)** The effective connectivity *J*_*eff*_ (see Equation 3) at timepoints halfway through the stim and the no-stim periods show vertical white bands due to the combined action of the non-linearity and adaptation. Thus, nonlinearity and adaptation dynamically modulate output gain and column sparsity of the effective connectivity, which reduces the spectral radius of the network’s Jacobian. **(F)** The eigenspectra of the full Jacobian matrices at these timepoints show that the SFA + STD system has more eigenvalues near the imaginary axis and a smaller spectral abscissa (leading real eigenvalue) than SFA-only at this timepoint. **(G)** We replicated the example simulation **(D)** with 25 random synaptic weight matrices similar to **(A)** and 25 random amplitude step stimuli. Importantly, we systematically varied the E:I ratio of the networks from 2:3 to 3:2 to determine whether E:I balance is important under four different adaptation conditions: no adaptation, SFA-only, STD only, and SFA + STD. Networks with no adaptation had increased chaos during stimulation (*p* = 0.035, two-tailed Wilcoxon signed rank test, median difference +0.46). Networks with SFA-only also had increased chaos (*p* = 0.0012, median difference +0.38). Networks with STD-only did not have a significant median change during stimulation (*p* = 0.31, median difference −0.08), and networks with SFA + STD did not have a significant change (*p* = 0.47, median difference 0.04). Notably, SFA + STD kept random, sparse, networks lacking E-I balance near the edge of chaos, even during random excitatory stimuli.

Here, the synaptic depression term *B* scales the columns of the structural connectivity matrix *W*, representing the reduced efficacy of presynaptic inputs. The activation function derivative Φ′(*h*) also appears as a column scaling term in the Jacobian, though it represents the postsynaptic sensitivity to input changes. This sensitivity is modulated by the adaptive SFA currents shifting the operating point *h*. Thus, adaptation and nonlinearity continuously reshape the network’s effective connectivity and stability (Figure 2). The multiple timescales of adaptation modulate input sensitivity through their interaction with nonlinearity; on a short timescale input sensitivity may be increased, but over extended timescales stability is controlled.

Synaptic depression *B* scales the connectivity matrix *W*. With differing scaling per column, the eigenvalues of *WB* will become concentrated near the center of the spectral disk (Rajan and Abbott, 2006). As a result, fewer eigenvalues will be near the edge of stability, reducing the complexity of the dynamics at the transition to chaos (Ipsen and Peterson, 2020). We hypothesize that multiple timescale SFA can counteract this concentration through homogenization. By suppressing the firing rate of highly active neurons, SFA could relieve STD and bring neurons out of saturation, increasing their gain Φ′(*h*). This regulation could homogenize the effective weight distribution across the network columns, reining in outlier eigenvalues and spreading the eigenvalue distribution more uniformly throughout the disk, improving both stability and complexity.

## 4 Discussion

Understanding how the brain maintains a regime of rich, complex dynamics near the edge of chaos despite being a highly recurrent, nonlinear network remains an active area of research. While structural constraints such as the precise balance of excitation and inhibition are thought to be critical, relying on static connectivity to maintain this balance may be insufficient. Indeed, finding a structural condition that constrains stability-threatening eigenvalue outliers while preserving low-dimensional dynamics remains difficult (see Figure 1; Harris et al., 2023). We propose that the solution over shorter timescales is likely dynamic rather than structural: multiple forms of adaptation may work in concert to regulate the network’s effective connectivity. While SFA alone may be insufficient to stabilize networks with supralinear activation functions, STD alone creates networks that are overly stable (Wu and Zenke, 2021). Therefore, we suggest that, in addition to slower intrinsic and homeostatic plasticity mechanisms that lead to self-organized criticality over extended timescales (Meisel and Gross, 2009; Páscoa dos Santos and Verschure, 2025; Wen and Turrigiano, 2024), multiple forms of adaptation help the brain operate near a homeostatic setpoint of chaos over shorter timescales and restore consistent dynamics when perturbed.

We suggest that SFA and especially the nonlinearity of STD and rectifying activation functions can be viewed as an explanation of time-varying effective connectivity. Data-driven linear time-varying (LTV) modeling is a successful and exciting area of research in epileptology (Li et al., 2021; Roy et al., 2025; Sritharan and Sarma, 2014). We believe that LTV models may be able to capture the modulation of effective connectivity by adaptation, a hypothesis that could be tested further using stimulation to probe the brain’s excitability. These approaches have the potential to influence the understanding of neurological conditions and could lead to the development of methods to measure the brain’s regulation of dynamical properties.

### 4.1 Adaptation regulates network excitability

We hypothesize that the interplay between presynaptic STD and multiple-timescale postsynaptic SFA regulates neuronal and synaptic excitability, keeping the brain on the edge of chaos. While STD provides strong nonlinear negative feedback, SFA can both decrease or increase excitability by reducing spike rate or by shifting the neuron away from saturation within the activation function. We propose that slow SFA timescales work in concert with STD to fine-tune this regulation, preventing over-stabilization. We showed that networks with both SFA and STD were most resilient to external perturbations (see Figure 2). Conversely, we hypothesize that insufficient adaptation may cause a loss of inhibitory dominance, allowing low-frequency eigenvalues of the Jacobian to approach the edge of chaos boundary. This transition could manifest as changes in the power of low frequency EEG, a phenomenon linked to the seizure onset zone and seizure susceptibility (Lundstrom et al., 2019, 2021; Lundstrom and Richner, 2023).

### 4.2 Data-driven modeling of effective connectivity

Data-driven dynamical models have the potential to quantify the level of stability or chaos in the brain. In epileptology, such models have identified “fragile” nodes, regions where small perturbations can destabilize the network (Li et al., 2021; Sritharan and Sarma, 2014). In anesthesiology, these tools have been applied to determine whether anesthetics stabilize or destabilize neural dynamics, though results depend on the modeling approach (Alonso et al., 2014; Dávila et al., 2025; Eisen et al., 2024; Solovey et al., 2015). Linear time-varying (LTV) models fit over short segments (∼0.5 s) suggest that propofol increases stability (Alonso et al., 2014; Solovey et al., 2015), while models which use longer segments (15 s) and time-delay embedding, HAVOK (Brunton et al., 2017), suggest that propofol decreases stability (Eisen et al., 2024). We suggest that this discrepancy is due to the multiplicative nonlinearity of STD and the difficulty observing adaptation from passive electrophysiological recordings. LTV models mitigate this through frequent refitting, but HAVOK may not handle poorly observed multiplicative nonlinearities well, as it performs best when the residual forcing term is sparse (Brunton et al., 2017). A linear parameter-varying (LPV) framework explicitly incorporating adaptation could bridge these findings, though more complex nonlinear base models make system identification and latent state estimation increasingly challenging.

When attempting to identify a system from electrophysiological recordings, stimulation can be used to help interrogate the underlying system (Hays et al., 2023; Kamali et al., 2020; Smith et al., 2020, 2022). We suggest that synaptic connectivity, SFA, and STD could potentially be distinguished by driving the system with a well-designed rich stimulus and by utilizing the fact that SFA is more linear than STD, and SFA is postsynaptic while STD is presynaptic. By analyzing the differential responses to varied stimulation patterns, it may be possible to estimate the contributions of these adaptation mechanisms to the effective connectivity.

### 4.3 Clinical relevance

Appreciating the regulatory role of adaptation extends the classic excitation-inhibition (E-I) balance hypothesis, a foundational concept in brain disorders involving cortical excitability. In the context of epilepsy, where unique access to intracranial electrophysiological recordings and stimulation is possible, there is a significant opportunity to develop advanced diagnostics based on quantifying adaptation and its response to external input, e.g., inside and outside the seizure onset zone. Bioelectronic medicine in the form of brain stimulation may be able to probe and alter adaptation to diagnose and treat disorders of neural excitability.

## Data Availability

The datasets presented in this study can be found in an online repository. Additional documentation, and all code to reproduce the data and figures, is available in the following repository: https://github.com/TomRichner/ConnectivityAdaptation.
